# Connecting proteins with drug-like compounds: Open source drug discovery workflows with BindingDB and KNIME

**DOI:** 10.1093/database/bav087

**Published:** 2015-09-16

**Authors:** George Nicola, Michael R. Berthold, Michael P. Hedrick, Michael K. Gilson

**Affiliations:** ^1^Skaggs School of Pharmacy and Pharmaceutical Sciences, University of California, San Diego, 9500 Gilman Drive, La Jolla, CA 92093, USA,; ^2^Department of Computer and Information Science, Konstanz University, 78457 Konstanz, Germany, and; ^3^Sanford Burnham Prebys Medical Discovery Institute, La Jolla, CA

## Abstract

Today’s large, public databases of protein–small molecule interaction data are creating important new opportunities for data mining and integration. At the same time, new graphical user interface-based workflow tools offer facile alternatives to custom scripting for informatics and data analysis. Here, we illustrate how the large protein-ligand database BindingDB may be incorporated into KNIME workflows as a step toward the integration of pharmacological data with broader biomolecular analyses. Thus, we describe a collection of KNIME workflows that access BindingDB data via RESTful webservices and, for more intensive queries, via a local distillation of the full BindingDB dataset. We focus in particular on the KNIME implementation of knowledge-based tools to generate informed hypotheses regarding protein targets of bioactive compounds, based on notions of chemical similarity. A number of variants of this basic approach are tested for seven existing drugs with relatively ill-defined therapeutic targets, leading to replication of some previously confirmed results and discovery of new, high-quality hits. Implications for future development are discussed.

**Database URL:** www.bindingdb.org

## Introduction

The last decade has seen a revolutionary rise in the open-source availability of experimental data connecting proteins, from humans and other organisms, with the drug-like small molecules that bind them (1). For example, BindingDB, the first public molecular recognition database (1–4) currently serves users with over a million quantitative protein-ligand interaction data, involving about 7000 proteins and 450 000 small molecules. This dataset represents a unified collection of quantitative binding data, which was assembled through curation of scientific journals and US patents by BindingDB, combined with ingestion of selected data from other important databases, notably ChEMBL (5), PubChem (6) and PDSP Ki (7). BindingDB and other publicly accessible medicinal chemistry resources now enable gene- and protein-oriented bioinformatics information to be systematically linked with drug discovery data, and are thus driving progress in interdisciplinary fields like systems pharmacology (8, 9).

BindingDB provides a range of useful browsing and search capabilities. As summarized in Table 1, these include the ability to select an article by journal name, volume and page number, and then retrieve its compounds and data in machine-readable format; or to quickly access binding data for a protein target of interest, based on its name, amino acid sequence, UniProt (10) ID, or PDB (11) ID. A number of BindingDB’s search capabilities are also accessible via URL search templates, which generate human-readable web-pages, and by RESTful webservices, which provide data in simple XML formats. The latter offer a straightforward API by which software running remotely can interactively access data from BindingDB. Alternatively, users can download parts of the BindingDB dataset, or its entirety, in several different formats, in order to enable entirely local execution of codes that use BindingDB data. However, this still requires software development, often through the use of scripting languages, to carry out flexible analyses of binding data or integrate it with the vast arrays of information contained in other public bioinformatics resources or in the user’s own local data archives.

**Table 1.** Commonly used BindingDB pages and information sources

Browse by journal article citation	bindingdb.org/bind/ByJournal.jsp
Browse by text name of protein target	bindingdb.org/bind/ByTargetNames.jsp
Search or browse by UniProt ID of target	bindingdb.org/bind/ByUniProtids.jsp
Search or browse by target sequence	bindingdb.org/bind/BySequence.jsp
Search or browse by PDB ID	bindingdb.org/bind/ByPDBids_100.jsp
Documentation of query template URLs	bindingdb.org/bind/SearchTemplates.jsp
Documentation of BindingDB RESTful services	bindingdb.org/Pathways/BindingDBRESTfulAPI.jsp
‘Find my compound’s target’ tool	bindingdb.org/bind/chemsearch/marvin/FMCT.jsp

Such challenges are increasingly addressed with flexible workflow environments for data analysis (12), such as Pipeline Pilot (13), Taverna (14), Kepler (15), Galaxy (16–18), Loni Pipeline (19) and KNIME (20). The latter is of particular interest here, as the computational chemistry community was an early adopter of the KNIME framework, and the open-source KNIME software is associated with a freely distributed desktop environment. The KNIME environment helps non-experts quickly develop and implement algorithms through the use of hundreds of individual ‘nodes’, each developed to perform a particular task, and allowing user configuration for custom data input and output. Nodes are developed in house at KNIME, as well as by developers in the open-source and for-profit communities, and are then made available as downloads. For example, there is a large selection of nodes for manipulation and analysis of chemical structures, such as calculation of molecular weight, Markush enumeration, evaluation of chemical similarity and other cheminformatics tasks [CDK (21), RDKit (22), Schrödinger (23, 24), ChEMBL (5), Open PHACTS (25), BioSolveIT (26)]. Thus, KNIME facilitates leveraging prior work by allowing users to flexibly combine nodes contributed by various developers in order to create new workflows.

The present study explores the combined usage of KNIME and the BindingDB dataset, through development and illustrative applications of a set of workflows, which make exclusive use of nodes that are freely available. We first describe two workflows which use KNIME nodes to interact with BindingDB’s RESTful web services. The first retrieves compounds known to bind a specific protein of interest, based on its UniProt ID; and the second retrieves proteins known to bind a specific compound of interest. These two workflows are envisioned as building blocks which can be incorporated into larger workflows that integrate protein- ligand binding data into broader bioinformatics analyses.

We also describe a set of more complex workflows, which implement the concept that a large database of binding data can provide hypotheses about the mechanisms of action of bioactive compounds and predict or explain the side effects of drugs (4, 27). Such hypotheses are based on the transitivity principle that a compound A which is chemically similar (28) to another compound B has increased likelihood of binding the same proteins as compound B (4, 27, 29–38). Accordingly, if A is found to have a biological activity, its mechanism might be due to binding the same proteins as compound B. Similarly, if A is a drug, it might generate side-effects by binding the same proteins as compound B. BindingDB’s Find My Compound’s Targets (FMCT) tool (Table 1) implements this idea, allowing users to enter one or more compounds and use BindingDB data to predict their protein targets. The present KNIME implementations of FMCT avoid network delays and possible confidentiality issues by doing the processing locally, using a downloaded representation of the necessary data from BindingDB. In addition to detailing the implementation of these workflows, we also evaluate their predictive performance by applying them to seven drugs recently identified as having unknown or uncertain mechanisms of action (32), and comparing the results to those from the Similarity Ensemble Approach (SEA) (35), which offers the same general functionality, but uses additional statistics in an effort to enhance the accuracy of the inferred compound–protein interactions. In addition to attempting to assign therapeutic targets to these drugs, we also seek to flag additional pharmacological activities, as these may suggest side-effects or drug-repurposing opportunities.

## Materials and methods

This section details the implementation of a set of KNIME workflows which process and use BindingDB data. The first subsection concerns two workflows which support common BindingDB database queries, and the second concerns several versions of the FMCT tool described above.

### Mining BindingDB for compounds and their protein targets

**Figure 1.**
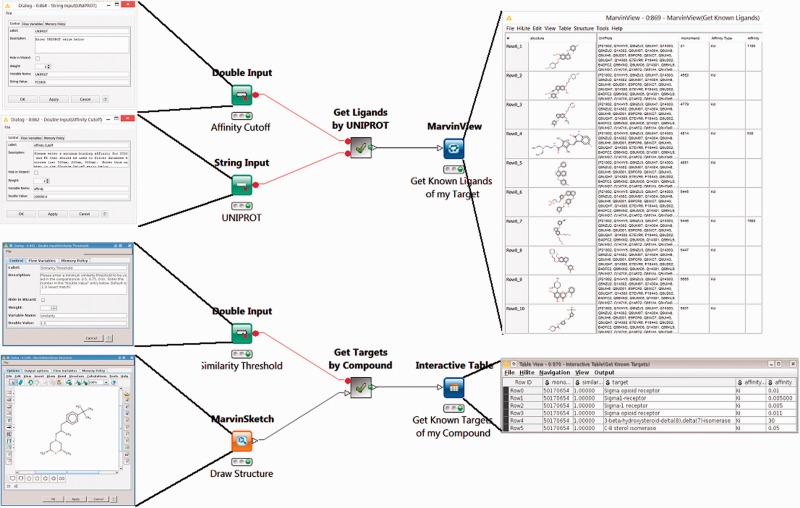
Two KNIME workflows to query the BindingDB server. Target2Compound (top) retrieves compounds from BindingDB that are known binders of a protein target of interest. Separate columns are created in the output for the compounds’ unique BindingDB monomerID, followed by its SMILES string, affinity types and affinity values. The collected results are stored in a table and can be written out to local disk. In this particular example, UNIPROT P21802 is entered into the dialog box, and various compounds are returned. The Compound2Target workflow (bottom) retrieves protein targets from the BindingDB database that are known to bind a particular compound of interest. Separate columns are created in the output for monomerID, Protein Target, affinity types (IC_50_ or K_I_) and affinity values (in nanomolar). The results are stored in a table which can be written out to local disk. In the given example, a sigma receptor binder is drawn in the MarvinSketch node, and the resulting output shows the expected targets, with their respective known affinities for the compounds hits.

The first two workflows use BindingDB’s web-services. The Target2Compound workflow (Figure 1, top) takes as input the UniProt ID(s) of one or more proteins, and returns compounds known to bind the corresponding protein(s) with an affinity (in nanomolar) above a user-specified threshold. The Compound2Target workflow (Figure 1, bottom) takes as input a single SMILES representation of a compound, or a drawing from an interactive KNIME chemical drawing node, contributed by ChemAxon, and returns all proteins for which an affinity measurement exists in BindingDB, along with the associated affinity data. The default mode is to search for exact compound matches, but one may also search by chemical similarity, measured on a scale of 0 to 1, where 1 is essentially an exact match.

The use of BindingDB web-services for these two workflows ensures that users receive up-to-date information from BindingDB, and takes advantage of built-in BindingDB query capabilities, notably chemical aware queries using the ChemAxon (39) toolkit. Both workflows utilize several KNIME nodes for handling URL based data. For example, the XML Reader node can upload a URL string, such as http://bindingdb.org/axis2/services/BDBService/getLigands By Uniprot?uniprot= in the case of Target2Compound, and then retrieve the resulting XML document. This document is then passed to a series of XPath nodes, which extract the appropriate data from the XML document.

### Predicting the protein targets of a compound

We implemented multiple versions of FMCT (see Introduction) as KNIME workflows. Each uses a different chemical similarity metric, but the overall procedures are essentially the same. In brief, the similarity of the query compound(s) is computed against every other compound in a reference database derived from BindingDB (see below). The compounds in the reference database whose similarity to the query compound is greater than a user-specified threshold then are ranked according to their similarity to the query compound, and the proteins known to be bound by these top-ranked reference compounds are identified. The initial list of top-scoring proteins identified in this way normally includes repeats, because several high-ranking compounds may bind one protein; such repeats are eliminated by merging protein hits with identical UniProt IDs. The results are then formatted into a report and presented to the user. Two sets of FMCT workflows were developed. One, designed for use with a single query compound, provides a full listing of the potential protein hits, with one protein per row. The second, designed for use with multiple query compounds, provides a reduced output table, in which each row lists one compound and its top three protein hits. As this condensed summary may not display some protein hits of interest, it is worth noting that users can modify the workflow to display more protein hits for each compound, or can make more extensive changes to provide alternative reporting formats.

These workflows do not use web-services, but instead use a KNIME Table Reader node to load a local data table of BindingDB data. The version of this table based on BindingDB’s current dataset contains approximately 670 000 rows of BindingDB data, with one binding measurement in each row, supplemented with multiple types of pre-computed compound fingerprints and chemical descriptors (see below). In effect, the data Table Reader encapsulate much of BindingDB in a single KNIME node. This approach maximizes performance and allows users to work entirely on their local computer, instead of having to upload possibly proprietary compounds to the BindingDB server. In order to speed FMCT calculations for use with large numbers of query compounds, we also created a reduced version of the full BindingDB table, which is filtered to include only a few representative compounds for each protein, as detailed below.

The FMCT method is based on the notion of chemical similarity (40–45), and we tested several different similarity metrics. Two of the metrics are based on chemical fingerprints (46–48). One simply applies the Tanimoto similarity (49) to the RDKit RDKit chemical fingerprint; the choice of this particular fingerprint from RDKit’s multiple options was made based on a suggestion from the RDKit team (Greg Landrum, personal communication). With a runtime of several minutes per query compound, this is easily the fastest FMCT workflow we built. The second fingerprint-based similarity metric was constructed from multiple fingerprint-based metrics, as follows. The current RDKit (22) and CDK (21) KNIME nodes support a total of 14 different binary compound fingerprints (Table 2). These can be compared with each other using three different formulas, Tanimoto, Cosine BitVector and Dice (28), for a total of 3 × 14 = 42 available similarity metrics. We applied all 42 methods to about 14 400 compound pairs drawn from known binders of the dopamine receptor and the thrombin receptor, used KNIME’s linear correlation node to generate a correlation table connecting each similarity scoring method, and then used KNIME’s correlation filter node to extract the 5 of 42 metrics that correlate least with each other (Table 3, and Supplementary Figure S5), as detailed in Results. We then implemented a version of FMCT in which the five similarity scores for a given pair of compounds are simply averaged with each other to yield an overall similarity metric.

**Table 2.** Chemical fingerprint types available in the CDK and RDKit packages in KNIME

CDK Standard
CDK Extended
CDK Estate
CDK PubChem
CDK MACCS
RDKit Morgan
RDKit FeatMorgan
RDKit AtomPair
RDKit Torsion
RDKit RDKit
RDKit Avalon
RDKit Layered
RDKit Pattern
RDKit MACCS

An enumeration of these types with each of the three similarity metrics available (Tanimoto, Cosine BitVector and DICE) results in 42 separate scoring methods.

**Table 3.** Similarity metrics and fingerprint types selected from the linear correlation filter (see text)

**Similarity metric**	**Fingerprint type**
KNIME Tanimoto	RDKit MACCS
KNIME Tanimoto	RDKit FeatMorgan
KNIME Tanimoto	RDKit Torsion
KNIME Cosine Bitvector	RDKit Morgan
KNIME Tanimoto	CDK PubChem

We also implemented versions of FMCT using a maximum common substructure (MCS) similarity metric (50–52) available in the KNIME MoSS MCSS Molecular Similarity node. This node required special treatment, however, as it produces an all-by-all comparison matrix within a set of compounds, rather than comparing two different sets of molecules. We addressed this by constructing a (parallel) chunking loop which iterates through pairs of molecules in two different columns by extracting each molecular pair as a row, transposing the columns, and computing the MCSS similarity of the pair. We then adjusted the output to provide similarity on a scale of 0–1, and used this in a separate FMCT workflow, which, again assigns similarity hits and candidate targets based on a user-specified similarity threshold.

Finally, even on a fast workstation, the MoSS MCSS Molecular Similarity node requires about an hour to scan one query compound across all 670 000 database entries (see below), so this approach becomes unwieldy if one wishes to run a large number of queries. The expectation that two compounds with a high MCS similarity should tend to also have a reasonably high fingerprint similarity led to implementation a fourth FMCT approach, which first runs the fast RDKit FP method, above, with some default or user-selected similarity threshold; and then re-ranks the resulting hits according to their MoSS MCSS similarity scores.

#### Building the BindingDB tables for the FMCT workflows

**Figure 2.**
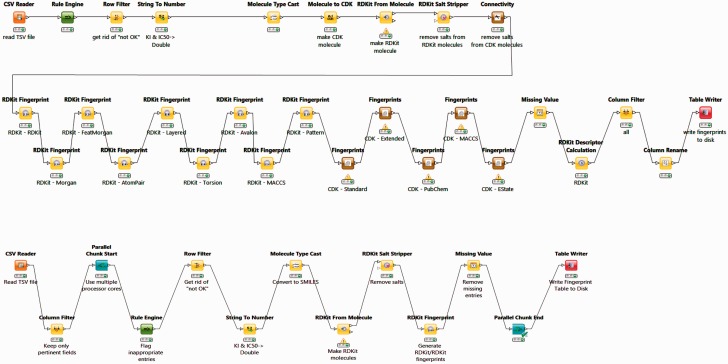
BDBBuilder workflows. These read in BindingDB data, filter for appropriate IC_50_ and K_I_ data, and convert SMILES strings to suitable molecular formats. The top workflow (BDBBuilder) generates RDKit and CDK molecules, multiple fingerprint types, and molecular descriptors. The bottom workflow (BDBBuilderFast) is a simplified version that only generates RDKit RDKit molecules/fingerprints. In both versions, the final node of the workflow writes the resulting table to disk.

The BindingDB tables used by the FMCT implementations described above were constructed using KNIME, as follows. A KNIME workflow, termed BDBBuilder (Figure 2), begins by reading in the standard, tab- separated-value file of all BindingDB data (http://tinyurl.com/o7ppdj9), which has one compound-target measurement per row; the version of this file used here has 1 043 465 rows. The workflow first filters out rows lacking IC_50_ or K_I_ measurements, or having affinity ranges, indicated by the use of ‘<’ or ‘>’ symbols in the input file. These filters reduce the number of rows to 677 825. Additional rows were removed if their compounds failed in the ‘RDKit From Molecule’ node’s MolSanitize function, or did not create a ‘CDK Fingerprint’ node’s Fingerprint string. This additional filter reduced the row count to approximately 670 000. Each data row was then supplemented with additional columns containing 2D chemical fingerprints and descriptors for the compound in the row. The fingerprints were generated using the following cheminformatics packages available as KNIME Community Contributions: CDK, Erlwood, Indigo and RDKit, each of which has its own fingerprint generation methods. In each case, the default settings of the nodes were used in the configuration. Of the nearly 50 fingerprint options provided, we selected a subset of 10 to retain, based on correlation analysis of associated similarity scores with the KNIME Correlation Filtering metanode. Thus, the 10 fingerprints are those which gave the least correlated similarity scores with each other, when used with a specific similarity metric. The 38 descriptors available from the RDKit package were similarly analyzed, and the 10 least correlated descriptors were added to the corresponding data rows. The output file is saved as a KNIME table, to be read in by a Table Reader node embedded in the FMCT workflows. Running the entire BDBBuilder workflow took about 3 h on an Intel Core i7 workstation. As only fingerprints, not descriptors, are used in the final workflows, we also developed a simpler version of this workflow, called BDBBuilderFast (Figure 2), which generates only RDKit RDKit molecules and fingerprints, and takes roughly 30 min to run.

In applications where one wishes to predict protein targets for more than several hundred compounds, using the full BindingDB tables (above) becomes time consuming. We therefore used a KNIME workflow, termed ClusteredData, to create a condensed version of the table, which includes only representative binding compounds for each protein target. We grouped compounds by their protein targets, and then applied a Tanimoto similarity method to their RDKit fingerprints, at a distance threshold of 0.8, to define clusters of compounds within each protein-specific group. Several representative, high-affinity compounds were then chosen as representatives from each cluster, and inserted into a KNIME table with the same structure as the full table described above. This procedure, which required about 120 h of CPU time on a desktop Intel Core i7 machine, resulted in a significant reduction of number of compounds, from 670 000 to approximately 21 000, and thus greatly expedited the FMCT studies. The consequences for the FMCT analysis are considered in Results.

## Results

This section presents the operation of the KNIME/BindingDB workflows described in Materials and Methods, and provides sample inputs and outputs. Readers are encouraged to download (tinyurl.com/jwqoulm and www.knime.org/example-workflows), test them with their own queries, and use them as starting points for their own applications. It is worth noting that workflows which use BindingDB web-services may be slowed unpredictably by network traffic, so extensive usage may benefit from using the downloaded BindingDB table (see Materials and Methods), as this enables queries to be executed on the user’s computer, while also affording maximum privacy. The setup instructions provided with the downloadable package of workflows provides suggestions for addressing issues that occasionally arise when running KNIME, such as server timeout errors or memory restrictions, through adjustments to the KNIME.INI configuration file.

### Finding compounds for targets and targets for compounds, based on known binding interactions

#### Operation of workflows

The Target2Compounds workflow (Figure 1, top) accepts a protein target, specified with a UniProt accession number, and returns compounds that this target binds with an affinity better than a specified cutoff. The default cutoff is 10 µM (K_I_ or IC_50_), but the user can optionally change this by modifying the Affinity Cutoff node (Figure 1, top left). Note that a UniProt accession number corresponds to a single protein chain, and this search can yield compounds binding multimeric proteins that contain one matching chain and other non-matching chains. As shown in the open dialog box (Figure 1, top left), the UniProt Primary Accession Number should be entered into the UniProt String Input node. The workflow is then executed by right-clicking on the MarvinView node at the end of the workflow. This triggers a query to BindingDB’s RESTful API webservice, which returns query results in XML format. Downstream nodes process the XML and parse it for individual entries, generating a tabular output with one row for each compound that binds the UniProt target. One may then display the results by selecting the MarvinView node’s Execute and Open Views option. Each row of the resulting table (Figure 1, top right) contains a SMILES string (transformed to a 2D structure in the MarvinView Node display) for the respective compound, the primary UniProt accession number of the desired target (along with possible obsolete UniProt accession numbers for the same chain), the compound’s BindingDB Monomer ID, a statement of the affinity type (K_I_ or IC_50_) and then the measured affinity (nM). Such tables can optionally be fed into subsequent chemical informatics nodes to look for common scaffolds or other patterns, combined with Target2Compounds outputs for other targets to look for overlaps or specificity patterns, or used as seeds for chemical database searches. The table of compounds can also be saved to disk in standard file formats, such as SDF (53) or comma-separated value (CSV), for subsequent use.

The Compound2Targets workflow (Figure 1, bottom) is the inverse of Targets2Compounds: it retrieves protein targets that bind a particular compound of interest. The default mode is to accept a compound via a MarvinSketch node (Figure 1, bottom left), which can be loaded with a SMILES string or by manual drawing, and to retrieve binding data for this exact compound, if it is found in BindingDB. The user may, optionally, replace the default exact-match search with a similarity search, by changing parameters in the node labeled Similarity Threshold (Figure 1, bottom left). Right-click and selecting Execute and Open Views on the Interactive Table node (Figure 1, bottom right) run the workflow and show the results. This workflow converts the query compound to a SMILES string and queries the BindingDB RESTful API webservice. The resulting hits in XML format are read, processed and parsed for individual entries. The output table provides a list of the compound hits, identified and sorted by their BindingDB Monomer IDs, along with all of BindingDB’s available data on the affinities of these compounds for their targets. As above, this table can be saved to disk in a number of standard formats, or can be piped into additional nodes added by the user for further data integration and processing.

#### Sample workflow usage and results

Mutations in the protein Fibroblast Growth Factor Receptor 2 are associated with breast cancer (54), so it is of interest to identify compounds which bind it. Using its UniProt ID, P21802, to initiate a query with the Target2Compounds workflow yields a substantial collection of binders (Figure 1, top). This workflow could be embedded within a larger KNIME workflow which would identify entire pathways of interest and use Target2Compounds to annotate them with information about known binders. Further, a user could browse for a particular pathway of interest on this page: http://bind ingdb.org/Pathways/pathways.jsp, click the BindingDB link that lists all the known proteins and associated UNIPROT IDs for that pathway, and then query each of these in the Target2Compound workflow. Although not currently set up to run a batch of targets, a simple KNIME Loop construct would speed this process.

**Figure 3.**
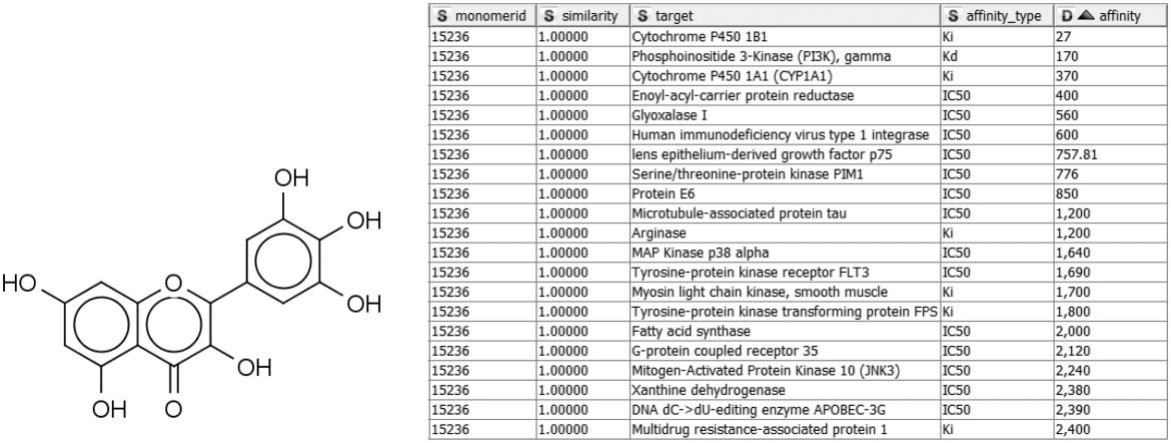
Sample query and results of Compound2Target workflow. Left: Query compound (IUPAC, 3,5,7-trihydroxy-2-(3,4,5-trihydroxyphenyl)-4H-chromen-4-one; SMILES, Oc1cc(O)c2c(c1)oc(-c1cc(O)c(O)c(O)c1)c(O)c2=O; InChI, 1S/C15H10O8/c16-6-3-7(17)11-10(4-6)23-15(14(22)13(11)21)5-1-8(18)12(20)9(19)2-5/h1-4,16-20,22H), which binds enoyl-acyl-carrier protein reductase with an IC_50_ of 400 nM. Right: Tabular output of Compound2Target workflow, showing significant potency (nM) of the same compound (similarity = 1) against multiple targets.

Alternatively, given a compound active against a target of interest, one may wish to identify other targets of the same compound, in order to study specific, polypharmacology, or mechanisms of toxicity. For example, the compound in Figure 3 may already be known to bind *P. falciparum* enoyl-acyl-carrier protein reductase with submicromolar IC_50_ (55), but a query with Compound2Targets reveals submicromolar potency against other, varied targets, including a human immunodeficiency virus integrase and protein kinase PIM1. Such information may be useful in selecting a compound as a research probe or as a starting point for medicinal chemistry optimization; or it may be taken as a caution regarding possible off-target effects of compounds in a specific structural class. Such applications are further developed in the following subsection describing the FMCT method.

### Using FMCT workflows to hypothesize new targets for compounds

#### Operation of target-finding workflows

These workflows predict possible new protein targets for compounds of interest. Straightforward applications of the concept of chemical similarity (28), they search the BindingDB dataset for compounds similar to a query compound, and suggest the targets of these compounds as potential targets of the query compound. In this way, the FMCT functionality, which was already available on the BindingDB web-site (bindingdb.org/bind/chemsearch/marvin/FMCT.jsp), can be run locally in KNIME and, optionally, incorporated into a user’s larger analytic workflows.

We implemented and tested four FMCT variants, which differ only in the similarity metrics they use. As detailed in Materials and Methods, one uses Tanimoto similarity with the RDKit RDKit chemical fingerprint; the second uses the average of five different fingerprint-based similarity metrics; the third uses a similarity metric based on the maximal common substructure (MCS); and the fourth is merely a re-ranking of the hits from the first metric based on MCS similarity. This takes advantage of the speed of the fingerprint method to identify similar compounds, while using the slower MCS similarity metric to highlight those similarities which also have the strongest substructure matches. It thus represents an attempt to obtain results similar to those from the pure MCS method, but at lower computational cost.

**Figure 4.**
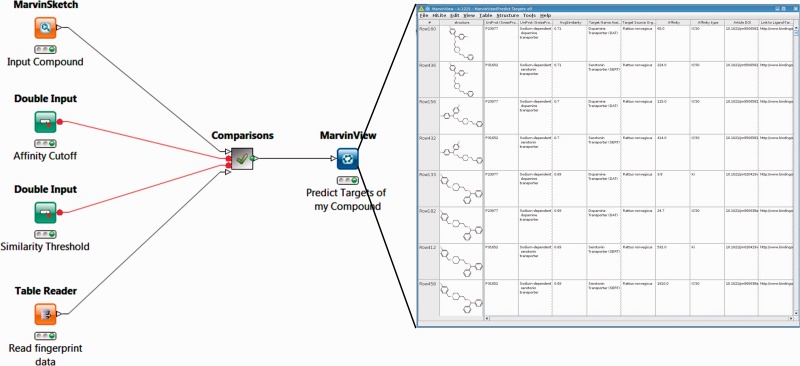
Workflow for FMCT methods designed for single query compounds. These workflows suggest what protein target(s) a compound of interest is likely to bind, based on the targets known to bind similar compounds. The MarvinSketch mode allows entry of the query compound. The Affinity Cutoff and Similarity Threshold nodes allow the user to set the stringency of these search parameters; affinity is specified in nM, and similarity on the usual 0–1 scale. When the run is complete, the green light will turn on below the MarvinView node, and one can right-click on it and choose ‘View: MarvinView’ to see the table of similar compounds, their similarities to the input compound and their associated targets. This figure depicts the workflow for FMCT performed by single RDKit fingerprint similarity. Workflows which also use the average of five different fingerprint similarity metrics, the RDKit RDKit results, sorted by MCS similarity, and a pure MCS similarity metric averaged, and single plus MCS resort (see main text) have very similar top-level views. The differences lie in the ‘Comparisons’ meta-node, which performs the calculations.

One set of FMCT workflows provides detailed results for a single query compound. For these, the generalized workflow for all four similarity metrics is shown in Figure 4; the differences among the four similarity metrics lie beneath this view, in the comparisons meta-node, which performs the similarity calculations. To run any of these workflows, the user double-clicks on the MarvinSketch Input Compound node and uses the resulting window to draw, paste or open a file with a small molecule compound of interest. Next, one can optionally modify the green Similarity Threshold and Affinity Cutoff nodes with desired values. Finally, one right clicks the MarvinView Predict Targets of My Compound node and selects Execute and Open Views; when the run is complete, the green light will turn on below this node. Right-clicking on it and choosing View: MarvinView shows the output table of similar compounds, their similarities to the input compound and their associated targets. If too many results are found, one may rerun the analysis with a higher (more stringent) compound similarity threshold, and/or a lower (more stringent) affinity cutoff (input as nanomolar).

A second set of these workflows provides less detailed target predictions for batches of query compounds, which are uploaded into an SDF Reader node. For the sake of a compact output, these workflows generate a table of results which does not show one suggested target per row, but instead lists one compound per row, with only the top three candidate targets in the associated columns. This allows for quick viewing of results for a large set of query compounds, but the incomplete target report can lead to missing an interesting target which does not make the top three. Users may therefore wish to adjust the output criteria to provide a more complete report. It should also be noted that processing a large batch of query compounds can become quite time-consuming, particularly for the relatively slow calculation using the MCS metric. As detailed in Materials and Methods, we sought to address this problem by using clustering to generate a smaller reference set with representative compounds; the results are summarized in the section ‘Accelerated target-finding with a reduced reference set’.

#### Application examples: hypothesizing new targets of bioactive compounds

We applied the single-compound FMCT workflows, with the four similarity metrics described above, to seven compounds previously identified (32) as drugs with unknown mechanisms of action. Previously, the SEA method was used to suggest one or more therapeutic targets for each of these compounds, and new experiments were done to determine whether they in fact bound the suggested targets. Here, we compare the top target hits from the present FMCT implementations (Table 4, columns 1–4) with the published SEA hits (Table 4, first SEA column). In addition, because the published SEA hits are only a subset of those suggested by the SEA algorithm, and because there may have been updates in the underlying databases since that research was done, we also looked more broadly at the SEA output for these seven drugs (Table 4, second SEA column) by running them again on the SEA website (http://sea.bkslab.org/search/). The prior SEA analysis of these compounds focused on discovering protein targets that might explain their therapeutic mechanisms. Here, we consider whether new target predictions made by FMCT could account not only for therapeutic mechanism but also for other known pharmacological activities, such as side-effects. We now consider each of the drugs in detail.

#### Cloperastine

**Figure 5.**
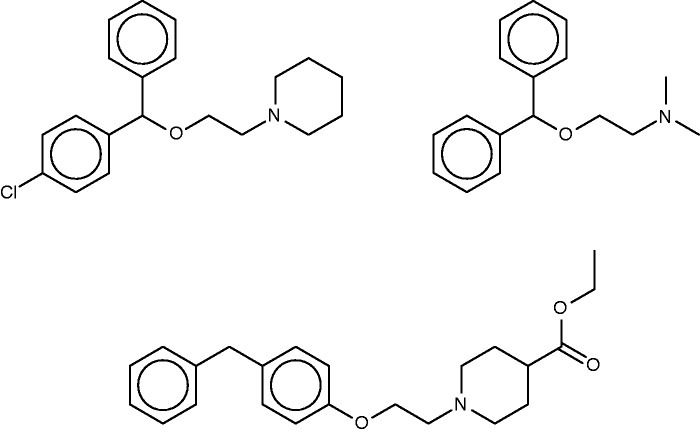
Cloperastine (top left), diphenhydramine (top right) and a known 1.8-µM inhibitor of leukotriene A4 hydrolase (bottom).

Cloperastine (Figure 5, top left) is a cough-suppressant which acts to suppress the CNS cough center (56). Discovered in studies of derivatives of the first-generation antihistamine diphenhydramine (Figure 5, top right) (57), it also possesses antihistaminic activity. The SEA study suggested and experimentally confirmed binding of cloperastine to the sigma receptor, and noted that this could explain its antitussive activity, based on the known relationship of activity at the sigma receptor type 1 to cough (58). (It is perhaps worth noting, however, that not only binding but also activation of the receptor would presumably be required to further verify this proposal.) Unsurprisingly, given the origin of cloperastine, it was also predicted and found to bind two subtypes of the histamine receptor. The SEA web-site similarly suggests sigma opioid receptor and one of the histamine receptor types as a top hits, but also assigns high scores (low *E*-values) to transporters of serotonin, dopamine and norepinephrine, as well as two neurotransmitter receptors, and, perhaps surprisingly, the enzyme leukotriene A4 hydrolase.

The FMCT results overlap strongly with those from SEA (Table 4a). Thus, all three of the fingerprint-based FMCT methods include the sigma receptor type 1 and histamine receptor type 1 among the top 10 hits (bold font). The latter was determined to be a hit for cloperastine based on this compound’s RDKit fingerprint similarity of 0.71 to diphenhydramine. Almost all of the additional hits from the SEA web-site also are hits for the fingerprint-based FMCT methods (Table 4a, asterisks). In addition, the unexpected enzyme hit, leukotriene A4 hydrolase, appears in only one of our methods, the pure MCS approach; the compound that led to this hit (Figure 5, bottom) has a very high MCS similarity score of 0.92 with cloperastine. An RDKit fingerprint similarity of these two compounds is only 0.41, which explains why it was not identified in any of our other methods. The higher similarity determined by MCS may be due to the likeness of individual substituents; one may imagine breaking cloperastine at the ether group and moving this, along with the connected piperidine ring, to the methylene joining the two phenyl groups. Whether the activities of these two compounds are similar will depend in large part on the 3D configuration and importance of these individual substituents in binding interactions with the protein target; readers are encouraged to decide for themselves whether the two compounds are indeed structurally similar.

**Table 4a.** Target hypotheses for cloperastine, a CNS-active cough suppressant, according to the present FMCT workflows (columns 1–4), a prior SEA paper (32) (column 4) and the SEA website http://sea.bkslab.org/search/ (column 5)

**RDKit similarity**	**Five averaged similarities**	**RDKit similarity**	**Pure MCS**	**SEA 2012 paper**	**SEA web**
		**MCS re-sort**			
0.77–0.71	0.71–0.68	0.64–0.63	0.92–0.85		8×10^−128^–10^−15^
*Sodium-dependent dopamine transporter *Sodium-dependent serotonin transporter **Sigma non-opioid intracellular receptor 1** **Histamine H1 receptor** Muscarinic acetylcholine receptor M5 *Muscarinic acetylcholine receptor M1 Muscarinic acetylcholine receptor M2 Muscarinic acetylcholine receptor M3 Muscarinic acetylcholine receptor M4 Potassium voltage-gated channel subfamily H member 2	*Sodium-dependent dopamine transporter *Sodium-dependent serotonin transporter *Norepinephrine transporter **Sigma non-opioid intracellular receptor 1** *Sodium-dependent noradrenaline transporter 5-hydroxytryptamine receptor 2 D(2) dopamine receptor Alpha-1A adrenergic receptor **Histamine H1 receptor** D(3) dopamine receptor	**Sigma non-opioid intracellular receptor 1** **Histamine H1 receptor** Muscarinic acetylcholine receptor M5 *Muscarinic acetylcholine receptor M1 Muscarinic acetylcholine receptor M2 Muscarinic acetylcholine receptor M3 Muscarinic acetylcholine receptor M4 Potassium voltage-gated channel subfamily H member 2 Solute carrier family 22 member 1 Solute carrier family 22 member 2	Acetylcholine-binding protein CHRNA7-FAM7A fusion protein Dopamine D3 receptor *Dopamine D4 receptor Dopamine Receptor D2 *Leukotriene A4 hydrolase Dopamine D1 receptor; DA D1 receptor Dopamine receptor D5 Neuronal acetylcholine receptor subunit beta-2 Sodium- and chloride- dependent GABA transporter 1	**σ receptor** **Histamine H1 receptor** Histamine H3 receptor	*Dopamine transporter *Serotonin transporter *Leukotriene A4 hydrolase *Sigma opioid receptor Histamine H3 receptor *Norepinephrine transporter Dopamine D4 receptor *Transporter Serotonin 4 (5-HT4) receptor *Muscarinic acetylcholine receptor M1

The SEA web-search used the ChEMBL v. 16 database, a binding affinity cutoff of 10 µM, and Scitegic ECFP4 fingerprints. In each case, outputs were grouped by unique Targets, and the top 10 hits are shown, with the best hits at the top, except for the SEA 2012 paper data, which are not ranked. FMCT Target names are drawn from UniProt (10). SEA web-site Target names are taken as reported. Bold indicates a match between FMCT and the published SEA results, and asterisks indicate matches between FMCT and SEA results from the SEA web-site. The range of similarity values for the listed target predictions are given under each column heading, where available, as generated by the corresponding methods.

More interestingly, one of the new FMCT hits gains substantial plausibility from external data. Thus, two FMCT methods suggest an interaction with the D(2) dopamine receptor, and a search of PubChem reveals a screening hit for cloperastine itself with the D(2) dopamine receptor (59). (Note that this datum is not in BindingDB, because BindingDB imports only confirmatory PubChem BioAssay data, and this is only a primary screening result.) Intriguingly, there are clinical case reports of dystonic reactions to cloperastine (60, 61), and dystonia is associated with agents that block the D(2) dopamine receptor. The SEA web-site similarly suggests the D4 dopamine receptor, though not D2, as a potential target of cloperastine. In summary, the straightforward chemical similarity methods implemented in FMCT yield a new target prediction which is encouragingly consistent with both *in vitro* and clinical data.

#### Nepinalone

**Figure 6.**
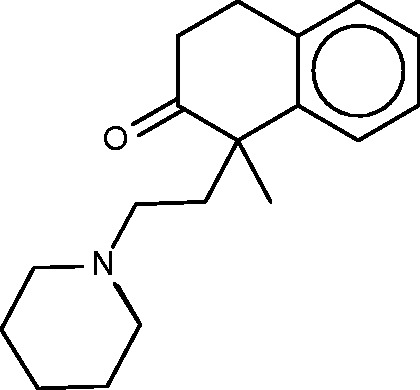
Nepinalone.

Nepinalone (Figure 6), another cough suppressant, is documented by a remarkably scant literature. The SEA method again identified the sigma receptor as a target of this drug, and confirmed binding *in vitro*, thus offering a potential therapeutic mechanism. All four FMCT methods similarly identified sigma receptor 1 as a top hit; and four other hits from the SEA web-site also appear in the FMCT results (Table 4b). For example, a steroid isomerase enzyme is highly ranked by SEA and two fingerprint methods in FMCT. It is thus encouraging that the sigma receptor and the isomerase have similar protein sequences, and that inhibitors of the isomerase bind the sigma receptor (62). The isomerase link might point to a novel side-effect mechanism for nepinalone, but this possibility is difficult to assess, due to the paucity of information on this drug.

**Table 4b.** Target hypotheses for nepinalone

**RDKit similarity**	**Five averaged similarities**	**RDKit similarity**	**Pure MCS**	**SEA **2012paper****	**SEA web**
		**MCS re-sort**			
0.95-0.64	0.68–0.54	0.63–0.58	0.74–0.70		3×10^−22^ – 4^−6^
*Sigma non-opioid intracellular receptor 1 D(2) dopamine receptor *3-beta-hydroxysteroid-Delta(8),Delta(7)-isomerase C-8 sterol isomerase D(3) dopamine receptor Vesicular acetylcholine transporter Aminopeptidase M 5-hydroxytryptamine receptor 2A *Nociceptin receptor Corticosteroid 11-beta-dehydrogenase isozyme 1	*Sigma non-opioid intracellular receptor 1 *3-beta-hydroxysteroid-Delta(8),Delta(7)-isomerase C-8 sterol isomerase D(2) dopamine receptor Vesicular acetylcholine transporter Sigma opioid receptor Nociceptin receptor *Histamine H3 receptor Muscarinic acetylcholine receptor M2 5-hydroxytryptamine receptor 2A	Vesicular acetylcholine transporter *Nociceptin/Orphanin FQ, NOP receptor Alpha-2A adrenergic receptor Alpha-2C adrenergic receptor Adrenergic Alpha2 Adrenergic Alpha2D Alpha-2B adrenergic receptor *Sigma non-opioid intracellular receptor 1 Glutamate-NMDA-MK801 5-hydroxytryptamine receptor 3A	Dopamine D3 receptor Dopamine Receptor D2 Glutamate receptor ionotropic, NMDA 2B Serotonin Receptor 1A *Sigma opioid receptor Vesicular acetylcholine transporter Sodium-dependent dopamine transporter Sodium-dependent noradrenaline transporter Sodium-dependent serotonin transporter Histamine H1 receptor	**σ receptor**	*Sigma opioid receptor *3-beta-hydroxysteroid-Delta(8),Delta(7)-isomerase Histamine *N*-methyltransferase *Histamine H3 receptor *Nociceptin receptor *Sigma-1 receptor Sodium channel protein type II alpha subunit Serotonin 2c (5-HT2c) receptor Histamine H1 receptor Muscarinic acetylcholine receptor M5

See Table 4a for details.

More generally, the top 10 targets suggested by the FMCT methods are more varied than those suggested for cloperastine, as they include additional enzymes and receptors. This variety may trace in part to the lower range of chemical similarities here than for cloperastine: the number of other compounds and hence candidate targets expands rapidly as one lowers the chemical similarity threshold. Although it is not clear that any particular similarity cutoff should be applied when one assesses the results of this type of analysis, it is clear that less confidence should be placed in target predictions based on lower similarity scores.

#### Clemastine

**Figure 7.**
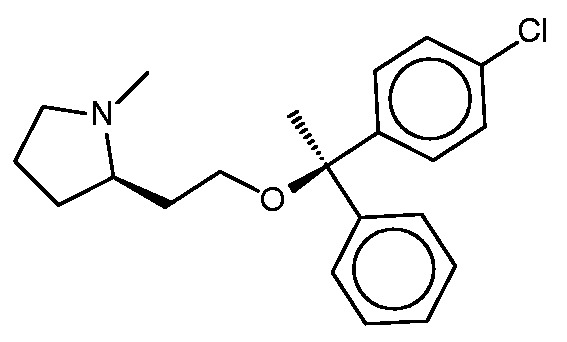
Clemastine.

Clemastine (Figure 7) is yet a third antitussive, which also has clinical antihistaminic and anticholinergic effects. The SEA study predicted and experimentally confirmed the sigma 1 receptor as a protein target of this compound. Perhaps surprisingly, then, the sigma 1 receptor does not appear among the top-ranked SEA web-site hits (Table 4c). Correspondingly, none of the FMCT methods identifies this target (Table 4c) either. There is significant overlap among the FMCT and SEA web-site predictions, particularly if one combines the subtypes of the muscarinic acetylcholine receptor (Table 4c); and the prediction of muscarinic activity is consistent with experimental evidence that clemastine promotes neuronal remyelination through activity at muscarinic receptors in oligodendrocytes (63).

**Table 4c.** Target hypotheses for clemastine

**RDKit similarity**	**Five averaged similarities**	**RDKit similarity**	**Pure MCS**	**SEA **2012 paper****	**SEA web**
		**MCS re-sort**			
1.00–0.70	0.92–0.58	0.92–0.64	1.00–0.79		10×10^−29^– 4^−27^
Perilipin-1 Perilipin-5 *Solute carrier family 22 member 1 Muscarinic acetylcholine receptor M1 Sodium-dependent dopamine transporter Sodium-dependent serotonin transporter Norepinephrine transporter Muscarinic acetylcholine receptor M2 D(2) dopamine receptor D(3) dopamine receptor	Perilipin-1 Perilipin-5 *Solute carrier family 22 member 1 Neuronal acetylcholine receptor subunit alpha-7 Neuronal acetylcholine receptor; alpha4/beta2 Nicotinic acetylcholine receptor alpha4/beta2/alpha5 *Neuronal acetylcholine receptor subunit alpha-10 Sodium-dependent dopamine transporter Sodium-dependent serotonin transporter Norepinephrine transporter	Perilipin-1 Perilipin-5 *Solute carrier family 22 member 1 Norepinephrine transporter Sodium-dependent serotonin transporter Sodium-dependent dopamine transporter Muscarinic acetylcholine receptor M1 Muscarinic acetylcholine receptor M2 Sodium-dependent dopamine transporter D(3) dopamine receptor	*Solute carrier family 22 member 1 Perilipin-1 Perilipin-5 Sodium channel protein type 2 subunit alpha D(3) dopamine receptor D(4) dopamine receptor D(2) dopamine receptor Sodium-dependent dopamine transporter Muscarinic acetylcholine receptor M2 *Histamine H1 receptor	**σ receptor**	*Solute carrier family 22 member 1 Muscarinic acetylcholine receptor M5 Muscarinic acetylcholine receptor M4 Serotonin 6 (5-HT6) receptor Cytochrome P450 2C19 Histamine H2 receptor *Histamine H1 receptor *Neuronal acetylcholine receptor protein alpha-10 subunit Synaptic vesicular amine transporter C-X-C chemokine receptor type 7

See Table 4a for details.

The present FMCT results also go beyond the results displayed by SEA, as FMCT reveals that clemastine itself has been shown to bind the lipid storage regulatory proteins perilipin 1 and 5 with 11 and 15 µM potencies, respectively (64). Although a literature search did not uncover any evidence that clemastine’s binding of the perilipins is clinically relevant, this activity could ultimately prove significant. The fact that these exact matches were not identified by web-based SEA tool may be attributed to its use of a 10-µM affinity threshold, which is not met by the 11 and 15 µM affinities of clemastine for the perilipins.

#### Benzquinamide

**Figure 8.**
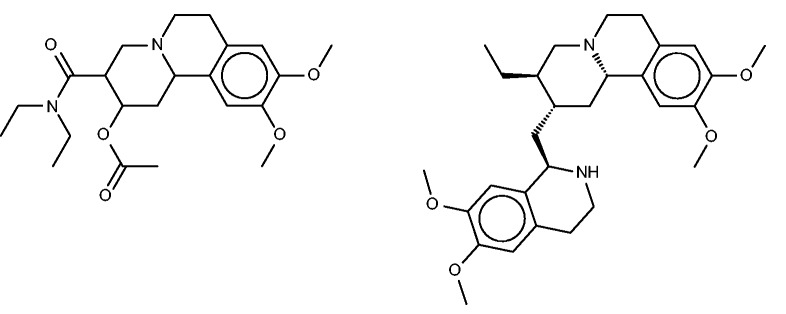
Benzquinamide (left) and emetine (right), a chemically similar compound identified by FMCT that is known to bind multidrug resistance protein 1.

Benzquinamide (Figure 8, left) is an obsolete antiemetic and anxiolytic drug (65). Although it is typically described as having antihistaminic and antimuscarinic activity (http://www.drugbank.ca/drugs/DB00767), the SEA analysis did not identify the histamine or muscarinic acetylcholine receptors as likely targets, and in fact went on to exclude these experimentally as high-affinity targets (32). Instead, the SEA analysis suggested the alpha-2 adrenergic receptor as a protein target, and this binding was confirmed experimentally. The similarity of known binders of the alpha-2 adrenergic receptors and binders of dopaminergic receptors then led to prediction and experimental confirmation that benzquinamide also binds to the dopamine (D2) receptor. Curiously, none of the FMCT methods suggests adrenergic receptors as targets, and these are also absent from the SEA web-site predictions. On the other hand, two of the fingerprint-based FMCT methods immediately yield the D(2) dopamine receptor as top hits (Table 4d), whereas the SEA web-site provides the similar D(1) dopamine receptor. A number of other top hits also are common between the SEA web-site and the FMCT results, but we were not able to uncover external confirmation or clinical correlates for these.

**Table 4d.** Target hypotheses for benzquinamide

**RDKit similarity**	**Five averaged similarities**	**RDKit similarity**	**Pure MCS**	**SEA 2012 paper**	**SEA web**
		**MCS re-sort**			
0.86–0.80	0.64–0.58	0.84–0.77	0.64–0.52		10×10^−77^–10^−02^
*Synaptic vesicular amine transporter *Dipeptidyl peptidase 4 Peptidyl-prolyl *cis–trans* isomerase FKBP1A Cytochrome P450 2D6 *D(1A) dopamine receptor **D(2) dopamine receptor** Multidrug resistance protein 1 COUP transcription factor 2 Nuclear receptor ROR-alpha *Putative hydrolase RBBP9	Synaptic vesicular amine transporter *Dipeptidyl peptidase 4 *Orexin receptor type 1 *Orexin receptor type 2 Cytochrome P450 2C9 Cytochrome P450 2D6 COUP transcription factor 2 Multidrug resistance protein 1 Nuclear receptor ROR-alpha *Putative hydrolase RBBP9	Synaptic vesicular amine transporter *Dipeptidyl peptidase 4 Multidrug resistance protein 1 COUP transcription factor 2 Nuclear receptor ROR-alpha *Putative hydrolase RBBP9 Steroidogenic factor 1 **D(2) dopamine receptor** *D(1A) dopamine receptor Melatonin receptor type 1B	Synaptic vesicular amine transporter *Dipeptidyl Peptidase IV (DPP-IV) Sodium-dependent dopamine transporter Norepinephrine transporter Integrin beta-3 Acetylcholinesterase Interstitial collagenase Collagenase 3 5-hydroxytryptamine receptor 4 Kallikrein-related peptidase 5 preproprotein	**Dopamine receptor (D2, D3, D4)** Adrenoceptor α2 receptor	*Synaptic vesicular amine transporter *Orexin receptor 1 *Orexin receptor 2 Melatonin receptor 1B *Putative hydrolase RBBP9 *Dipeptidyl peptidase IV Small conductance calcium-activated potassium channel protein 1 Carbonic anhydrase XIV *Dopamine D1 receptor Carbonic anhydrase VII

See Table 4a for details.

All three fingerprint-based FMCT analyses furthermore predict that benzquinamide may bind multidrug resistance protein 1, also known as P-glycoprotein. Emetine, the similar compound in BindingDB which led to this prediction is shown in Figure 8 (right). Given this prediction, we did a literature search, which yielded apparent confirmation in a paper (66) stating that ‘Benzquinamide inhibits P-glycoprotein-mediated drug efflux and potentiates anticancer agent cytotoxicity in multidrug resistant cells’. It is not immediately clear why the SEA method did not make this prediction, given the chemical similarity of benzquinamide to emetine, and the fact that emetine binds the multidrug resistance protein with an affinity better than 10 µM.

#### Lobenzarit

**Figure 9.**
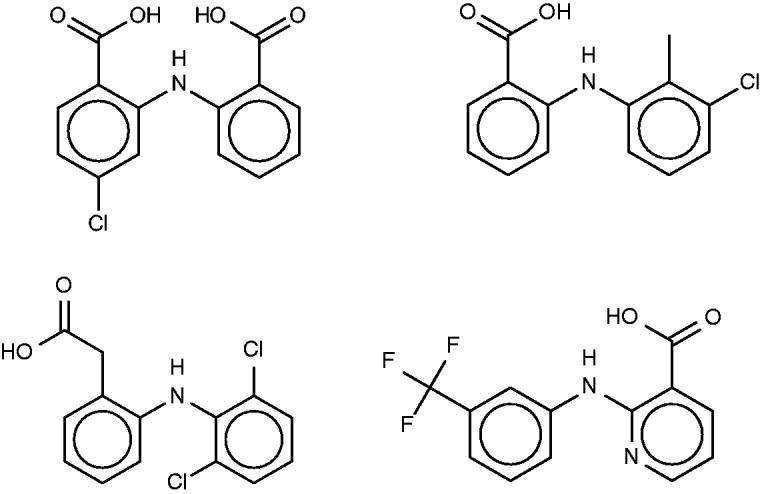
Lobenzarit (top left), tolfenamic acid (top right, a known inhibitor of phospholipase A2), diclofenac (bottom left) and niflumic acid (bottom right).

The drug lobenzarit (Figure 9, left) is an immunomodulator (67) which is used to treat rheumatoid arthritis. The prior SEA analysis suggested and experimentally confirmed inhibition of COX-2 by lobenzarit, leading to the suggestion that COX-2 inhibition may be the therapeutic mechanism of this drug. Somewhat surprisingly, COX-2 does not rise to the top 10 of the SEA web-site’s target rankings. However, it does appear, listed as prostaglandin G/H synthase 2, in two of the FMCT rankings, along with the closely related enzyme prostaglandin G/H synthase 1 (Table 4e). Two hits from the SEA web-site list, both alpha-keto reductases, do appear in all four of the FMCT lists, but there is otherwise little concordance between the two methods.

**Table 4e.** Target hypotheses for lobenzarit

**RDKit similarity**	**Five averaged similarities**	**RDKit similarity**	**Pure MCS**	**SEA 2012paper**	**SEA web**
		**MCS re-sort**			
0.91–0.77	0.76–0.68	0.82–0.70	0.85–0.80		10×10^−156^– 2^−20^
*Aldo-keto reductase family 1 member C3 *Aldo-keto reductase family 1 member C2 Hexokinase Androgen receptor Cysteine protease ATG4B Phospholipase A2 Anthranilate phosphoribosyltransferase 17-beta-Hydroxysteroid Dehydrogenase 5 (17-beta-HSD5, AKR1C3) NAD-dependent protein deacetylase sirtuin-1 Carbonic anhydrase 1	*Aldo-keto reductase family 1 member C3 *Aldo-keto reductase family 1 member C2 Hexokinase Androgen Receptor (AR) Cysteine protease ATG4B Phospholipase A2 Androgen receptor Aldo-keto reductase family 1 member C1 **Prostaglandin G/H synthase 2** Prostaglandin G/H synthase 1	*Aldo-keto reductase family 1 member C3 *Aldo-keto reductase family 1 member C2 Hexokinase Androgen receptor Cysteine protease ATG4B Phospholipase A2 Anthranilate phosphoribosyltransferase 17-beta-Hydroxysteroid Dehydrogenase 5 (17-beta-HSD5, AKR1C3) NAD-dependent protein deacetylase sirtuin-1 Carbonic Anhydrase IX	*Aldo-keto reductase family 1 member C2 *Aldo-keto reductase family 1 member C3 Aldo-keto reductase family 1 member C1 Androgen Receptor Prostaglandin G/H synthase 1 **Prostaglandin G/H synthase 2** Cysteine protease ATG4B Phospholipase A2 17-beta-Hydroxysteroid Dehydrogenase 5 (17-beta-HSD5, AKR1C3) Carbonic Anhydrase IX	**COX-2**	*Aldo-keto reductase family 1 member C2 *Aldo-keto-reductase family 1 member C3 Beta-ketoacyl-ACP synthase III Glutamate receptor ionotropic kainate 1 UDP-galactose 4-epimerase Aldo-keto reductase family 1 member C1 Interleukin-8 Hydroxycarboxylic acid receptor 2 Calcium-activated potassium channel subunit alpha-1 Liver glycogen phosphorylase

See Table 4a for details.

All four FMCT methods also predict binding of lobenzarit to a phospholipase A2, a prediction not made by SEA. The phospholipase inhibitor responsible for this prediction, tolfenamic acid (Figure 9, top right), is clearly similar to lobenzarit (Figure 9, top left). Tolfenamic acid is actually an existing non-steroidal anti-inflammatory drug, and hence a known COX (cyclooxygenase) inhibitor, in accord with the SEA and FMCT hits on this target. BindingDB’s additional contribution is that tolfenamic acid is a 7.7-µM inhibitor of phospholipase A2. This result derives from a counterscreen (PubChem AID 588400), rather than a primary screen. Interestingly, phospholipases A2 have been implicated in a variety of inflammatory and autoimmune disorders, including rheumatoid arthritis, one of lobenzarit’s indications. We therefore tested lobenzarit in the same phospholipase inhibition assay that turned up tolfenamic acid as an inhibitor, while also re-testing tolfenamic acid. Contrary to the prediction based on molecular similarity, lobenzarit showed no significant inhibition at concentrations up to 300 µM, whereas tolfenamic acid was found to have an IC_50_ of 20 µM, similar to the previously reported value of 7 µM; see Supplementary Figure S6. This result clearly argues against the target hypothesis. On the other hand, it seems equally clear that one would not want to miss uncovering the chemical similarity of lobenzarit to tolfenamic acid evident in Figure 9; and it is still of interest to consider whether lobenzarit might inhibit another phospholipase involved in inflammatory pathways. Some further support for this concept is afforded by recent co-crystal structures of the similar (Figure 9) anti-inflammatory agents diclofenac [2b17 (68)] and niflumic acid [1td7 (69)] with a snake phospholipase A2 (UniProt PA2A3_NAJSG) having 51% sequence identity with human phospholipase A2 (UniProt PA21B_HUMAN).

#### Cyclobenzaprine

**Figure 10.**
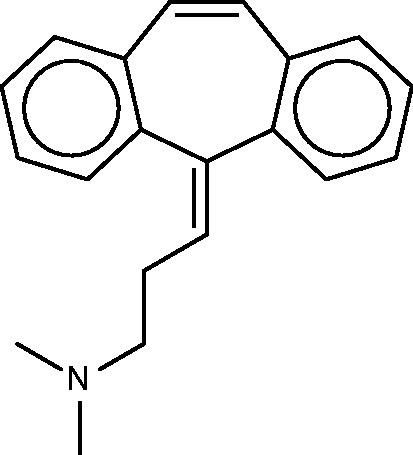
Cyclobenzaprine.

Cyclobenzaprine (Figure 10) is a centrally acting muscle relaxant. Its known activity at the 5-hydroxytryptamine (serotonin) receptor 2A might explain its therapeutic activity (http://www.drugbank.ca/drugs/DB00924), and it may also act at adrenergic receptors (70). The SEA analysis further predicted and confirmed binding to the M1, M2 and M3 muscarinic acetylcholine receptors, and suggested that this hitherto unknown interaction could play an important role in cyclobenzaprine’s therapeutic activity. (It is perhaps worth mentioning that, although modulation of muscarinic receptors might explain changes in smooth muscle tone, it seems less clear that it could account for the relaxation of skeletal muscle, for which cyclobenzaprine is used.) The SEA analysis also suggested and confirmed binding at the H1 histamine receptor. Both the present FMCT methods (Table 4f) and the SEA web-server provide very similar target predictions, especially if one lumps together different subtypes of each receptor. All of the FMCT prediction methods also suggest several new targets, but we were unable to find confirmatory data in the literature; some of these could be of interest to follow up experimentally.

**Table 4f.** Target hypotheses for cyclobenzaprine

**RDKit similarity**	**Five averaged similarities**	**RDKit similarity**	**Pure MCS**	**SEA 2012 paper**	**SEA web**
		**MCS re-sort**			
0.85–0.85	0.77–0.77	0.79–0.79	0.91–0.68		10×10^−46^ – 3^−03^
D(2) dopamine receptor *5-hydroxytryptamine receptor 2A **Histamine H1 receptor** 5-hydroxytryptamine receptor 1A *Muscarinic acetylcholine receptor M5 **Muscarinic acetylcholine receptor M1** **Muscarinic acetylcholine receptor M2** **Muscarinic acetylcholine receptor M3** *Muscarinic acetylcholine receptor M4 Norepinephrine transporter	*Muscarinic acetylcholine receptor M5 *5-hydroxytryptamine receptor 2A 5-hydroxytryptamine receptor 1A **Histamine H1 receptor** **Muscarinic acetylcholine receptor M1** **Muscarinic acetylcholine receptor M2** **Muscarinic acetylcholine receptor M3** *Muscarinic acetylcholine receptor M4 Norepinephrine transporter Sodium-dependent noradrenaline transporter	D(2) dopamine receptor 5-hydroxytryptamine receptor 2A *5-hydroxytryptamine receptor 2C 5-hydroxytryptamine receptor 6 *Muscarinic acetylcholine receptor M5 Norepinephrine transporter 5-hydroxytryptamine receptor 1A **Histamine H1 receptor** **Muscarinic acetylcholine receptor M1** **Muscarinic acetylcholine receptor M2**	Adenosine A3 Receptor **Histamine H1 receptor** Sodium-dependent noradrenaline transporter Sodium-dependent dopamine transporter Aldose reductase Protein skinhead-1 *Alpha-1A adrenergic receptor D(2) dopamine receptor Sigma non-opioid intracellular receptor 1 D(1A) dopamine receptor	**Muscarinic M1 receptor** **Muscarinic M2 receptor** **Muscarinic M3 receptor** **Histamine H1 receptor**	*Histamine H1 receptor *Alpha-1a adrenergic receptor *Muscarinic acetylcholine receptor M4 Serotonin 2b (5-HT2b) receptor *Serotonin 2c (5-HT2c) receptor *Muscarinic acetylcholine receptor M5 Alpha-1b adrenergic receptor Alpha-2b adrenergic receptor Pleiotropic ABC efflux transporter of multiple drugs Histamine H2 receptor

See Table 4a for details.

#### Nefopam

**Figure 11.**
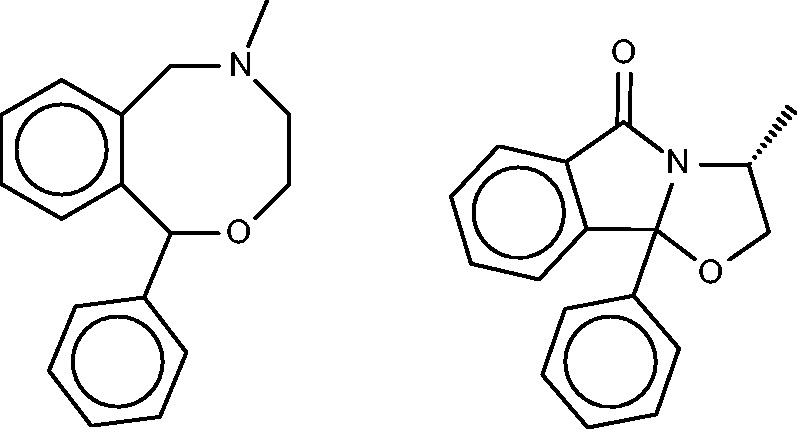
Nefopam (left) and a 40-µM inhibitor of HIV-1 reverse transcriptase inhibitor (right) identified by the pure MCS metric as similar to nefopam.

Nefopam (Figure 11, left) is a centrally active analgesic, for which the SEA publication suggested and confirmed binding to serotonin receptors. Allowing less compelling *E*-values furthermore turned up the dopamine transporter, which was also confirmed as a hit, along with the serotonin and norepinephrine transporters. The present FMCT methods (Table 4g) predict primarily muscarinic receptor and histamine receptor binding, in agreement with the SEA web- server, but we were unable to find literature bearing on these predictions. It might be of concern that the top 10 FMCT hits did not include the serotonin and norepinephrine transporters, but these do appear a little lower in the lists; e.g. positions 20–22 for the single RDKit fingerprint method, where they appear with chemical similarity scores of 0.61.

**Table 4g.** Target hypotheses for nefopam

**RDKit similarity**	**Five averaged similarities**	**RDKit similarity**	**Pure MCS**	**SEA 2012 paper**	**SEA web**
		**MCS re-sort**			
0.72–0.72	0.59–0.59	0.66–0.66	0.88–0.88		2×10^−19^– 4^−01^
Potassium voltage-gated channel subfamily H member 2 *Muscarinic acetylcholine receptor M5 *Histamine H1 receptor Muscarinic acetylcholine receptor M1 Muscarinic acetylcholine receptor M2 Muscarinic acetylcholine receptor M3 Muscarinic acetylcholine receptor M4 Solute carrier family 22 member 1 Solute carrier family 22 member 2 Multidrug and toxin extrusion protein 1	*Muscarinic acetylcholine receptor M5 *Histamine H1 receptor Muscarinic acetylcholine receptor M1 Muscarinic acetylcholine receptor M2 Muscarinic acetylcholine receptor M3 Muscarinic acetylcholine receptor M4 Potassium voltage-gated channel subfamily H member 2 Solute carrier family 22 member 1 Solute carrier family 22 member 2 Multidrug and toxin extrusion protein 1	Muscarinic acetylcholine receptor M4 *Muscarinic acetylcholine receptor M5 *Histamine H1 receptor Multidrug and toxin extrusion protein 1 Muscarinic acetylcholine receptor M1 Muscarinic acetylcholine receptor M2 Muscarinic acetylcholine receptor M3 Potassium voltage-gated channel subfamily H member 2 Solute carrier family 22 member 1 Solute carrier family 22 member 1	HIV-1 Reverse Transcriptase	Dopamine transporter Serotonin transporter Norepinephrine transporter Serotonin receptor 2A Serotonin receptor 2B Serotonin receptor 2C	Dopamine D5 receptor *Muscarinic acetylcholine receptor M5 Dopamine D1 receptor *Histamine H1 receptor Adenylate cyclase type V Dopamine transporter Alpha-1a adrenergic receptor Sigma opioid receptor Norepinephrine transporter Serotonin 2c (5-HT2c) receptor

See Table 4a for details.

#### Comparison of fingerprint versus MCS similarity in FMCT

Inspection of Table 4 reveals that the fingerprint-based FMCT methods tend to give similar target predictions, whereas the pure MCS method often generates distinctive results. It is instructive to examine the specific example of cloperastine, for which pure MCS suggests leukotriene A4 hydrolase as a protein target, but the fingerprint methods do not. The similar compound in BindingDB which generates this MCS similarity hit is shown in Figure 5 (bottom), where it may be compared with cloperastine itself (Figure 5, top left). Perhaps not surprisingly, the RDKit fingerprint similarity between these two compounds is only 0.41. The pure MCS similarity metric also yields distinct target suggestions for cyclobenzaprine (aldose reductase) and nefopam (HIV-1 reverse transcriptase; see Figure 11, right). The fingerprint approaches appear to correlate somewhat more strongly with the web-based SEA results than does the pure MCS approach, probably because the similarity metric underlying SEA is a type of chemical fingerprint, rather than one based on MCS. Overall, the pure MCS approach provides distinctive target suggestions that may merit attention. However, it is far more time- consuming than the fingerprint methods, so we tested a hybrid approach in which MCS similarities were computed for only the most similar fingerprint hits, and the fingerprint hits were re-ranked by MCS similarity. Although the resulting method is faster, it does not succeed at highlighting the interesting hits found by pure MCS, as evident by inspection of Table 4.

#### Accelerated target-finding with a reduced reference set

**Figure 12.**
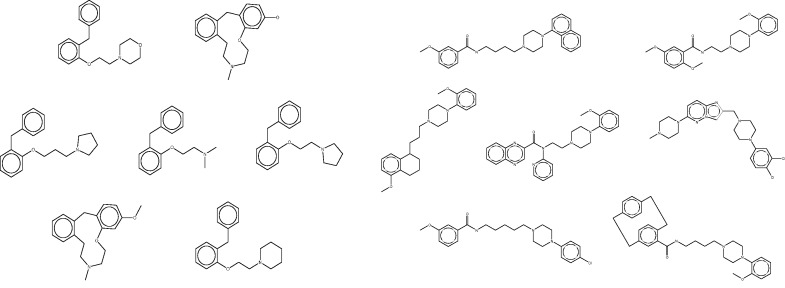
Representative clustered data sets of Dopamine D4 binders. On the left, all seven compounds from a single cluster set. On the right, 7 of 126 compounds clustered in another set. Although high similarity is evident in the cluster set on the left, it is apparent that several compounds are not as similar to each other in the cluster set on the right. In each case, a representative member of the cluster is selected at random for use in the reduced data set.

The fingerprint-based FMCT methods require a few minutes on a single commodity computer to scan a query compound against the full 670 K compound reference set, whereas the MCS method requires over an hour. These calculations thus can become time-consuming in applications where one wishes to process multiple compounds, such as a commercial compound catalog. One solution is to use parallel processing to reduce the wall-clock time, but it is also of interest to reduce the overall computational requirements. We conjectured that similar results might be returned if compounds were scanned not against the full reference set, but instead against a reduced set generated by clustering all compounds in the full set based on RDKit fingerprint similarity, and keeping only one representative compound from each cluster (see Materials and Methods). The reduced reference set contained only 21K compounds, a roughly 30-fold reduction, and the FMCT workflows were accordingly accelerated; for example, the pure MCS workflow requires only a few minutes per compound when used with the reduced reference set. However, the increased speed came at the cost of a significantly reduced ability to recover important hits. For example, although the full reference set allowed FMCT to rediscover 13 of the 20 confirmed target hits reported in the SEA paper for the seven drugs (as well as several plausible new targets), the present reduced reference set turned up only four. To illustrate the performance of the clustering technique, and perhaps an explanation for the lack of predictions with the reduced set, we highlight two cluster sets from a single target, Dopamine D4 (Figure 12). As is apparent by looking at these compound structures, the diversity within the cluster sets varies considerably, as does the value of selecting a representative member of each set at random.

### Packaging and dissemination of workflows and data tables

The main user workflows described above, and the data tables they use, are available for download at the websites for BindingDB (tinyurl.com/jwqoulm). They also can be accessed through the KNIME desktop, via the KNIME Explorer, under EXAMPLES, as described here: http://www.knime.org/example-workflows, and filtering for ‘BindingDB’. In all cases, the left-most nodes displayed in the workflow accept user input, and the right-most nodes collect and display output. The output is displayed in a KNIME table by default, but can be also saved in a variety of formats (CSV, SDF, MOL, etc.) or channeled to other workflows/nodes for follow-on analysis. The same repositories also provide a number of related utility workflows, such as the BDBBuilder and ClusteredData workflows, used to produce BDBBuilder Table and ClusteredData Table, respectively. These may be used to update the data tables used by the FMCT workflows as new BindingDB data become available on the BindingDB Download page.

## Discussion

### Integration of BindingDB into KNIME workflows

The integration of BindingDB with KNIME workflows sets the stage for incorporation of BindingDB data into a wider range of applications, which can take advantage of the growing ecosystem of KNIME nodes and workflows supporting chemical informatics, bioinformatics and data analytics (https://tech.knime.org/knime-community) (71). Various use-cases may be envisioned, for example:

- Target2Compounds may be used to annotate biomolecular signaling pathways with information about available small molecule modulators, as a step to moving from systems biology to systems pharmacology.
- FMCT workflows can suggest mechanistic targets for compounds discovered to be active in high-throughput phenotypic assays, such as ones in which cellular changes induced by millions of compounds are detected by microscopy and categorized by automated image analysis.
- The Compound2Targets and FMCT workflows may find application screening commercial catalogs for known and potential activities.
- The FMCT workflow may be used to generate reports of possible side-effects of compound series being considered for development into candidate drugs.

Further applications will become possible as additional computational and analytic methods are encapsulated into KNIME nodes. For example, at least two companies offer KNIME nodes with ligand-protein docking functionalities (www.schrodinger.com/knimeworkflows, http://www.molsoft.com/gui/knime.html).

The workflows described here exemplify two modes of accessing BindingDB data. In the first, KNIME nodes access BindingDB’s web-services on-the-fly. This always uses the most up-to-date results and does not require downloading and processing large files. In the second, the full BindingDB dataset is processed into a KNIME table which resides on the user’s computer. This avoids possible network and server delays during use, is appropriate for large-scale data access and allows the user to ensure reproducibility of his or her query results. It also maximizes privacy and allows for customized queries that go beyond what is offered in the web-services. It is worth noting that users wishing to adjust the criteria for winnowing BindingDB’s large dataset into the local KNIME table can do this by modifying the workflows used to build the table, BDBBuilder, BDBBuilderFast or CondensedData. Moreover, a full BindingDB data dump is available for download, so users can set up their own local installations of the entire database.

### Predicting protein targets for compounds with BindingDB data

The present study also illustrates the emergent value of a large enough database of protein-ligand binding data, as a tool to predict protein targets for compounds of interest. Valuable data from a published application of the SEA methodology (32) provided a platform to evaluate several different FMCT implementations. Although the SEA and FMCT studies are more anecdotal than statistical, they nonetheless provide a useful initial picture of how the various methods perform. Overall, the FMCT methods provide excellent recovery of the target proteins correctly identified by SEA. Although FMCT does miss several targets correctly identified by SEA, it also identifies valid or highly plausible targets that do not appear to be highly ranked by SEA. These differences between FMCT and SEA may result from a combination of factors, including differences between the reference databases used, the application of a 10-µM affinity cutoff in the web-based SEA application, and the different ways that chemical similarity is computed and used to predict targets for query compounds. Thus, in using the web-based SEA application we selected the ChEMBL database; but ChEMBL does not include some compound-protein affinity data present in BindingDB. It is thus worth noting that the SEA web-page also allows one to use alternative databases, including KEGG (72) and WOMBAT (73). In addition, although SEA takes a holistic statistical approach to associating compounds with potential targets, FMCT is based on simple pairwise comparisons of compounds, and the two approaches naturally yield different, and seemingly complementary, target predictions.

Of the similarity metrics tested here, the fast RDKit fingerprint method performed as well as or better than the more complex alternatives. However, the MCS similarity metric occasionally provided interesting results distinct from those provided by the fingerprint methods. Because the MCS similarity metric is relatively slow to compute, we tried using MCS to rerank a reduced set of targets already highly ranked by a fingerprint method, but, although this saved computer time, it did not recover all the interesting hits provided by the full MCS method. We also experimented with accelerating FMCT by using a much smaller reference dataset, which was constructed by clustering all compounds in the full reference set according to chemical similarity, and extracting a single representative compound from each cluster. However, this approach led to substantially reduced recovery of validated and plausible targets for the query compounds examined here. The results might be improved in future work by allowing a more generously sized reference set in order to reduce the diversity of the compounds in each cluster, and potentially by using a different clustering method.

For general users, it is worth pointing out that the core fingerprint-based FMCT functionality is also freely accessible to users via interactive pages at the BindingDB website, which does not require any familiarity with KNIME (www.bindingdb.org/bind/chemsearch/marvin/FMCT.jsp). This web implementation yields similar, but non-identical results, as it employs a different set of chemical fingerprints, generated with JChem (74), to compute chemical similarity.

For users interested in KNIME, the present workflows provide a starting point for development of novel implementations of FMCT, and for incorporation of this functionality into more comprehensive workflows. For example, one might employ other chemical similarity metrics, such as ones based on molecular shape (75–78) or three-dimensional pharmacophore models based on available protein-ligand co-crystal structures. It is also interesting to envision broader integrations, such as a merger of FMCT predictions with natural language processing of the scientific literature to automatically generate annotations of genes and pathways with compound and targeting predictions; or the use of machine-learning techniques already available in KNIME nodes to craft more powerful cheminformatics analysis tools. Thus, the use of KNIME to analyze BindingDB data and to integrate it with additional data types potentiates a range of useful biomedical and translational applications.

## Supplementary Data

Supplementary data are available at *Database* Online.

Supplementary Data
