# Supplementary material for: Connecting proteins with drug-like compounds: Open source drug discovery workflows with BindingDB and KNIME
Source: Database (Oxford). 2015 Sep 16;2015:bav087. doi: 10.1093/database/bav087 (PMC4572361; doi:10.1093/database/bav087)
Supplement: Supplementary Data [file supp_2015_bav087_index.html]

Connecting proteins with drug-like compounds: Open source drug discovery workflows with BindingDB and KNIME — Supplementary Data 

# Connecting proteins with drug-like compounds: Open source drug discovery workflows with BindingDB and KNIME

## Supplementary Data

files

- Supplementary Data - docx file
