# Supplementary material for: Connecting proteins with drug-like compounds: Open source drug discovery workflows with BindingDB and KNIME
Source: Database (Oxford). 2015 Sep 16;2015:bav087. doi: 10.1093/database/bav087 (PMC4572361; doi:10.1093/database/bav087)
Supplement: Supplementary Data [file supp_bav087_Supplemental_Information_v4.docx]

Supplemental Information

## BindingDB KNIME Setup Instructions

The BindingDB team has designed several KNIME workflows that emulate existing web-based functionalities, including compound and target data retrieval and the “Find My Compound’s Targets” tool. In addition to the KNIME workflows, the installation package comes with necessary BindingDB datasets, and can be used directly within a standalone KNIME Desktop installation. Supplemental Information Table 1 is a summary of what is included in this package.

1. To get the KNIME functionality to work properly, you first need to download and install the latest version of KNIME Analytics Platform from here: <http://www.knime.org/downloads/overview>
2. Of the different versions for each platform (Supplemental Information Figure 1), you may download the barebones installation, which is listed first, or the version with all free extensions (larger file), which is listed second. If you choose the first option, then you will need to manually install a set of KNIME extensions for the present workflows to operate, as described in Step 3. If you choose the version that includes all the free extensions, skip to Step 4.
3. After installing and opening KNIME, from the Help menu, select Install New Software; install the RDKit KNIME Integration nodes, Marvin Chemistry nodes, Network Mining nodes, KNIME XML processing nodes, KNIME Virtual nodes, and KNIME Nodes to create KNIME Quick Forms, as shown in a few representative screenshots (Supplemental Information Figure 2).
4. Once KNIME and all the required extensions are installed, you can import the workflows you want to use by one of the following two options:

**Option 1 -**  **Downloading from BindingDB web site.**

- - 1. At the bottom of this page <http://goo.gl/lMLq04>, you can download a ZIP package with all the KNIME workflows. Note: the single ZIP file is a package of all the individual workflows (also ZIP files) aggregated together. You need only to unzip the main package, and retain the underlying workflows in their ZIP format.
    2. To load workflows in KNIME, select File, Import KNIME Workflow, Archive File, and browse for one of the workflows (as a ZIP file) listed above, as “Source”. For the “Target”, you can use the default destination location “LOCAL”.

**Option 2 – Downloading from KNIME EXAMPLES server**

1. In the KNIME EXPLORER window, right-click the EXAMPLES header and login to the server. Once the examples are displayed, navigate and expand “050_Applications”, and locate the workflows that have names preceded by “XXXXXX_BindingDB_...” (Supplemental Information Figure 3).
2. These workflows can be individually or multiply selected and dragged to your local workspace under heading LOCAL.
3. In the KNIME Explorer window on the left-hand side of the KNIME desktop, expand the LOCAL (Local Workspace) header and double-click the workflow to load it. Once a workflow is loaded, you will see individual instructions for it in a yellow annotation box, as illustrated below. Generally, the left-hand nodes need to be configured with input structures or values, and the right-hand nodes display the final results; these can be executed by right-clicking and selecting “Execute and Open Views”, or using the menu bar button as shown in the screenshot below. Note that the FMCT workflow can take some time to load the BindingDB data table and then process it. Progress bars below most nodes are updated continuously during the run. However, some nodes, such as the Comparisons node in FMCT, are actually containers for a more complicated hidden workflow. These do not show progress bars, but you can double-click on the node to see the hidden details, and these nodes will display progress bars (Supplemental Information Figure 4).
4. Finally, if running any of these workflows results in Server Timeout Errors, the user is advised to add the following line to the KNIME.INI configuration file: -D.knime.url.timeout=100000. If you experience slowdown due to memory issues, you can try increasing the allowed memory usage by changing the entry for -Xmx512m  to -Xmx1g, for example, or higher, as described here: <https://tech.knime.org/faq#q4_2>. You may also consider to change the option -XX:MaxPermSize=256m to 1g or higher depending on your available memory. Note that your main memory must be at least the sum of both values. Also, the multiple blue “Warn” comments that sometimes display during runtime can be ignored; KNIME defaults to verbose commenting.

## Correlation Matrix

Correlation analysis was performed on nearly 50 chemical similarity metrics available in KNIME. Here, we show a representative submatrix showing correlations among the 9 metrics available in the RDKit package (Supplemental Information Figure 5). For this analysis, ten compounds were chosen as representatives of ten structural clusters of dopamine receptor binders.

## Phospholipase A2 Protocol and Dose Response Curves

For the Phospholipase A2 (PLA2) enzyme assay, test compounds lobenzarit and tolfenamic acid were pre-incubated with 200 nM PLA2 enzyme in 3 uL of assay buffer containing 20mM TRIS-HCl pH 8.0, 2mM CaCl2, 1mM DTT for 15 minutes at range of test concentrations in black, 1536-well assay plates (Greiner Cat# 782076). Following the 15 minute pre-incubation of test compounds with enzyme the PLA2 reaction is initiated by the addition of 3 uL of assay buffer containing 40 uM of the substrate NBD-C6-HPC (Life Technologies Cat# N-3786). Assay plates were incubated in the dark for 45 minutes after which time the plates read on a PerkinElmer Viewlux uHTS Microplate Imager at 485 nm excitation and 535 nm emission wavelengths. Supplemental Information Figure 6 shows representative dose response curves for A. tolfenamic acid and B. lobenzarit in the PLA2 enzymatic assay.
